# Genetic associations in beta-lactam allergy

**DOI:** 10.1186/2045-7022-4-S3-P68

**Published:** 2014-07-18

**Authors:** Jean-Louis Gueant, Antonino Romano, Jose Antonio Cornejo-Garcia, Rosa-Maria Guéant-Rodriguez, Francesco Gaeta, Miguel Blanca

**Affiliations:** 1 Inserm and university of Lorraine, France; 2 Complesso Integrato Columbus, UCSC-Allergy Unit, Italy; 3 Carlos Haya Hospital of Malaga, Allergy Service, Spain; 4 Université de Lorraine and Inserm, Inserm U 954 Nutrition-Génétique-Exposition, France

## 

Immediate reactions to beta-lactams (BLs) are the most common allergic reactions against drugs and can be life-threatening. The genetic risk factors influencing these reactions are poorly known, except those, which were previously identified as predictors of atopy and inflammation. Two polymorphisms of nucleotide-binding oligomerization domain 2 (NOD2), a gene associated with chronic inflammatory bowel diseases, influence the risk of BL allergy (BLA), in Italy and Spain. G>A at --308 of TNFA, a key gene of inflammation, is a significant independent predictor of the primary risk of BLA, in central Italy. TNFA is part of an extended haplotype HLA-A1-B8-DR3-DQ2, suggesting a potential implication of drug recognition by major histocompatibility complex. Several studies in Europe, US and China converge in showing also that immediate-type allergic reactions to BLs are predicted by genes, which influence IgE production, IL-13, IL-4 and IL4RA. The combination of the less frequent allele of the IL13 R130Q polymorphism with any of the predominant homozygous genotypes of 3 polymorphisms of IL4RA was more significantly associated with the risk of BL allergy than any polymorphism considered alone, in Italy. In contrast, in Spain, IL13 and IL4RA had no epistatic influence and only IL4RA I50V and Q551R were predictors of BL allergy (39). These gene variants were also associated with IgE against prevalent allergens and total IgE, respectively. The association of IgE against house dust mite with BL allergy could take part in the high frequency of BL allergy in Spain, where the exposure to house dust mite is higher than in colder European countries. A polymorphism in the gene promoter of the high-affinity receptor for IgE (FcεR1β) is a predictor for increased IgE sensitization to cephalosporins, in exposed health care workers from Korea. Very recently, we have performed a genome wide-association study (GWAS) in immediate-type BL allergic subjects and matched controls from Spain, with replication of significant associations in an Italian population. The results of this GWAS highlight the importance of drug presentation in the genetic susceptibility of BLA. In conclusion, the predictors of immediate allergic reactions to betalactams reflect the importance of genetic and environmental factors through mechanisms related to IgE production and antigen presentation.

